# The opportunity of using durum wheat landraces to tolerate drought stress: screening morpho-physiological components

**DOI:** 10.1093/aobpla/plad022

**Published:** 2023-05-05

**Authors:** Latifa Chaouachi, Miriam Marín-Sanz, Zayneb Kthiri, Sameh Boukef, Kalthoum Harbaoui, Francisco Barro, Chahine Karmous

**Affiliations:** Laboratory of Genetics and Cereal Breeding (LR14 AGR01), National Institute of Agronomy of Tunisia, Carthage University, 1082 Tunis, Tunisia; Department of Plant Breeding, Institute for Sustainable Agriculture-Spanish National Research Council (IAS-CSIC), 14004 Córdoba, Spain; Laboratory of Genetics and Cereal Breeding (LR14 AGR01), National Institute of Agronomy of Tunisia, Carthage University, 1082 Tunis, Tunisia; High Institute of Agronomy of Chott Mariam, Sousse University, Chott-Mariem 13, Sousse 4042, Tunisia; Higher School of Agriculture of Mateur, Carthage University, 7030 Route de Tabarka, Tunisia; Department of Plant Breeding, Institute for Sustainable Agriculture-Spanish National Research Council (IAS-CSIC), 14004 Córdoba, Spain; Laboratory of Genetics and Cereal Breeding (LR14 AGR01), National Institute of Agronomy of Tunisia, Carthage University, 1082 Tunis, Tunisia

**Keywords:** Assessment, field capacity, tolerance, *Triticum turgidum* ssp. *Durum*, water stress

## Abstract

Local genetic resources could constitute a promising solution to overcome drought stress. Thus, eight (8) durum wheat landraces and one improved variety were assessed for drought tolerance in pots under controlled conditions. Three water treatments were tested: control (100 % of the field capacity (FC)), medium (50 % FC) and severe (25 % FC) stress. The assessment was carried out at the seedling stage to mimic stress during crop set-up. Results showed that increased water stress led to a decrease in biomass and morpho-physiological parameters and an increase in antioxidant enzyme activities. Severe water stress decreased the chlorophyll fluorescence parameters, relative water content (RWC) and water potential of the investigated genotypes by 56.45, 20.58, 50.18 and 139.4 %, respectively. Besides, the phenolic compounds content increased by 169.2 % compared to the control. Catalase and guaiacol peroxidase activities increased 17 days after treatment for most genotypes except Karim and Hmira. A principal component analysis showed that the most contributed drought tolerance traits were chlorophyll fluorescence parameters, RWC and electrolyte conductivity. Unweighted pair group method with arithmetic mean clustering showed that the landraces Aouija, Biskri and Hedhba exhibited a higher adaptive response to drought stress treatments, indicating that water stress-adaptive traits are included in Tunisian landraces germplasm.

## Teaser

This study aimed to select contrasting water stress-tolerant durum wheat genotypes from a Landraces gene pool, using morpho-physiological and biochemical traits. The results showed that landraces have much greater genetic tolerance aptitude than the improved variety to mitigate water stress constraints. Indeed, the Landraces Aouija and Hedhba were identified as tolerant to water stress. Besides, the improved variety Karim is the most sensitive to water stress conditions. Our data showed that chlorophyll fluorescence parameters, relative water content, and electrolyte conductivity are the most reliable traits to screen for drought tolerance.

## Introduction

Durum wheat (*Triticum turgidum* ssp. *Durum*) is a crucial cereal crop, constituting the most widely worldwide staple food, providing high carbohydrate, protein content and calories for the human diet (Enghiad *et al*., 2017; Liu *et al*., 2017). The actual wheat yield should be increased by 70 % to meet the global population needs expecting to reach 9.7 billion people in 2050 (Alexandratos and Bruinsma, 2012; United Nations, 2022).

Drought constitutes the main limiting abiotic stress factor for wheat productivity. It affects around half of the world’s wheat-sown fields of 230 million hectares (Pfeiffer *et al*., 2005). In those areas, the wheat yield might decrease by 50–90 % (Olivares-Villegas *et al*., 2007). This situation will become more serious in the future due to climate change scenarios. In fact, predictions of climate change involve rises in average temperatures of 2.6 and 4.8 °C by 2065 and 2100, respectively, associated with more frequent periods of drought and heatwaves (Allan *et al*., 2021).

Improvement of wheat yields is a complex and challenging objective mainly for cropping in dry areas where water is a limiting factor (Jia *et al*., 2017). Water availability is critical at all plant growth stages from early plant growth to grain filling (Helaly *et al*., 2017). In fact, stress events during vegetative growth significantly decreased wheat grain yield (Abid *et al*., 2018*a*; Sehgal *et al*., 2018; Yu *et al*., 2018; Cohen *et al*., 2021). Yield losses due to drought stress can reach 25 % under 40 % of water deficit and 32 % in the case of no irrigation at reproductive and grain-filling growth stages (Daryanto *et al*., 2016, 2017; Anwaar *et al*., 2020). Moreover, the effects of water deficiency are dependent on both the severity and duration of stress (Bista *et al*., 2018; Laxa *et al*., 2019).

Various studies described the trend of changes in morphological, physiological and biochemical traits (Boussakouran *et al*., 2019; Jallouli *et al*., 2019; Othmani *et al*., 2019; Pour-Aboughadareh *et al*., 2020; Gholamin and Khayatnezhad, 2020; Ayed *et al*., 2021; Tefera *et al*., 2021; Quagliata *et al*., 2023). Drought negatively affects plant morphological and physiological functions such as biomass, shoot and root growth (Al-Maskri *et al*., 2016; Widuri *et al*., 2018). Water stress reduced photosynthetic pigments, leaf area (LA) and water potential, and led to excessive synthesis of reactive oxygen species (ROS) (Bhuiyan *et al*., 2019).

Osmotic adjustment constitutes an important mechanism to lower osmotic potential and thus maintain leaf water status and cellular functions under low soil water content (Abid *et al*., 2018*a*, *b*; Alghory, 2019; Haider *et al*., 2020; Abeed *et al*., 2021). Osmotic adjustment is obtained through the synthesis and accumulation of different water-soluble organic compounds such as proline and soluble sugars (Yu *et al*., 2019; Wang *et al*., 2019, 2020). Proline accumulation in plants is related to water stress tolerance (La *et al*., 2019; Moreno-Galván *et al*., 2020), and it is the most biochemical trait of drought tolerance (Singh *et al*., 2014). Lowering osmotic potential can help water uptake (Rani *et al*., 2022) and maintain cellular turgor (Abid *et al*., 2018*a*; Soni *et al*., 2021). This is particularly important since metabolic processes appear to be more closely linked to cell turgor than leaf water potential (Leuschner *et al*., 2019; Soni *et al*., 2021).

The ROS family includes different members such as singlet oxygen ${(}^{1}O_{2})$, superoxide $\left(O_{2}^{-}\right)$, hydrogen peroxide (H_2_O_2_) and hydroxyl radical (OH^−^) (Zhang *et al*., 2015). H_2_O_2_ causes several damages to cellular structures (Nazir *et al*., 2020; Asgher *et al*., 2021). In addition, H_2_O_2_ is a stress signal involved in increasing photosynthetic and durum wheat plant growth under abiotic stress condition (Asghar *et al*., 2021).

To quench the excess of ROS generated in cells, plants exhibit various enzymatic and non-enzymatic antioxidants and increasing the accumulation of osmo-protectants and compatible solutes (Sekmen *et al*., 2014), which help the plants to reach oxidative homeostasis (Rather *et al*., 2020*a*, *b*; Asgher *et al*., 2021). H_2_O_2_ mitigates abiotic stress through upregulating the activity of antioxidant enzymes, defence proteins and transcription factors (Sun *et al*., 2016; Nazir *et al*., 2020; Asgher *et al*., 2021).

The present study aimed to identify drought-tolerant durum wheat landraces using morpho-physiological traits and enzymatic activities as screening parameters. The drought stress mechanism was also investigated for a better understanding and management of drought stress.

## Materials and Methods

### Plant material and growth conditions

The used plant material was constituted of nine durum wheat genotypes (*T. turgidum* ssp. *Durum*) including eight landraces (Mahmoudi, Hmira, Swabaa Aljia, Jneh Khotifa, Chili, Biskri, Hedhba and Aouija) from GenBank Tunisia and one improved variety (Karim) from central seed mutual society of Tunisia.

The seeds of the different genotypes were disinfected with 5 % sodium hypochlorite for 10 min, then rinsed three times with distilled water. The seeds were germinated on Wattman filter paper and placed in Petri dishes for 24 h. The germinated seeds were transplanted into plastic pots (2 L) containing a mixture subtract of peat:perlite (2:1; V/V) at the rate of three plants per pot. No additional fertilization has been added since the peat used contains the main nutrients the plant needs (nitrogen (N), phosphorus (P) and potassium (K)) in the following proportions: 14:10:18. A completely randomized experimental design was adopted with three replicates for each genotype. The plants were grown under controlled conditions of 21 ± 2 °C (night/day), a photoperiod of 16/8 h and an average humidity of 60 %.

After 15 days of growth (Z11) (Zadoks *et al*., 1974), water stress treatments were applied as 100 % of the field capacity (FC) (Control), 50 % FC and 25 % FC. Water irrigation treatments were monitored by weighing pots every 2 days. The stress was applied over a period of 18 days (Z14).

### Morpho-physiological traits

A set of morpho-physiological traits were assessed. The aerial part length (APL) was measured by a caliper from the collar to the tip of the longest leaf according to the method described by Morard (1986). The fourth LA was measured using ImageJ software (version 1.46r) (Wayne Rasband, NHI, USA).

Dry matter rate (DM) (%) was recorded for aerial part of the plants. After measuring initial fresh weight (FW), they were placed in an oven at 80 °C for 48 h. The obtained material was weighted as dry weight (DW). The DM (%) was obtained according to the present formula:


$$\begin{array}{l} \text{DM }\left(\%\right)=\frac{\text{DW}}{\text{FW}}100 \end{array}$$

Canopy temperature was measured by infrared thermometry of the Helect (China) type. Besides, chlorophyll fluorescence components such as initial fluorescence (F_0_), maximum fluorescence (*F*_m_), variable fluorescence (*F*_v_), maximum fluorescence efficiency (*F*_v/m_) and leaf photosynthetic capacity (*F*_v/o_) were evaluated using an Opti-Science30 (Opti-Sciences Inc., USA).

The relative water content (RWC) (%) was evaluated for the flag leaf according to Turner (1986). Thus, the leaves were cut at the base of the blade, weighed immediately to determine the FW, then placed in Vial tubes filled with distilled water and stored in the dark. After 24 h, the samples were weighed again to obtain the turgor weight. These same samples were then dried in an oven at 55 °C for 48 h before being reweighed to determine the DW. The calculation was made according to the following formula:


$$\begin{array}{l} RWC\;\left(\%\right)=\left(\;\frac{FW-DW}{TW-DW}\right)100 \end{array}$$

The plant water potential (Ψ_w_) was set up according to the method of Canny (1997) using a pressure chamber (PMS Instrument Co., Corvallis, OR, USA). The electrolyte conductivity (EC) was determined according to the method described by Ben Dkhil and Denden (2012). Indeed, leaf samples were washed with distilled water to remove surface-adhered electrolytes and cut into discs of uniform size. The flag leaf discs were put in closed test tubes containing 10 mL of deionized water and incubated at room temperature (25 °C) for 24 h. Subsequently, the electrical conductivity of the solution (EC1) was recorded. Samples were then autoclaved at 120 °C for 20 min and the final electrical conductivity (EC2) was obtained after cooling the solution to room temperature. The EC was calculated as EC1/EC2 and expressed as a percentage.

The chlorophyll Index (CI) was determined using SPAD 502 chlorophyll meter, Konica Minolta brand, Zhejiang. The readings are provided in units called SPAD (Soil Plant Analysis Development).

### Biochemical traits

Five biochemical traits were measured as proline content (PC), soluble sugars content (SSC), H_2_O_2_ content (H_2_O_2_), malondialdehyde content (MDA) and phenolic compounds. First, the leaf PC was determined using the method of Bates *et al*. (1973). The plant sample (100 mg) is treated with 40 % methanol and then heated to 85 °C for 1 h. Then 1 mL of extract is added to a mixture of distilled water, acetic acid and ninhydrin. Second, the SSC in leaves was also measured according to the method of Shields and Burnett (1960). Thus, 100 mg of leaf were ground with 10 mL of ethanol (80 %) and incubated in a water bath for 30 min at 70 °C. After cooling, the extract is centrifuged at 6000 rpm for 10 min. Subsequently, 50 μL of the supernatant are added to 5 mL of anthrone (2 %) and 2.45 mL of ethanol (80 %). Absorbance was measured at 640 nm. The H_2_O_2_ content was determined by spectrophotometry according to Sergiev *et al*. (1997). Indeed, 100 mg of fresh plant material is ground in 1 mL of a 0.1 % TCA (trichloroacetic acid) solution. The ground material is then centrifuged at 12 000 rpm for 15 min at 4 °C. Then, a volume of 0.5 mL of the supernatant is incubated in the presence of 1 mL KI (1 M) and added to 0.5 mL of potassium phosphate buffer (KH_2_PO_4_/K_2_HPO_4_; 10 mM; pH 7). The mixture is then homogenized and incubated for 5 min. The optical density is determined at 390 nm. Under conditions of oxidative stress, plants exhibit a chain of lipid peroxidation ultimately leading to the production of MDA, a reactive aldehyde capable of reacting with 2-thiobarbituric acid. MDA extraction and assay were performed according to the method of Ksouri *et al*. (2007). The last biochemical measure is the total phenolic compounds or polyphenols (Ph.C), which were determined using the Folin–Ciocalteu reagent according to the method of Singleton *et al*. (1999).

### Antioxidant enzyme activity

Antioxidant enzyme activities were measured as guaiacol peroxidases (GPX) and catalase activity (CAT). The activity of GPX was measured according to the method reported by Egley *et al*. (1983). In fact, 500 mg of fresh plant material were ground in a mortar containing a volume of 5 mL of a phosphate buffer (50 mM; pH = 6.5). The ground material obtained is then centrifuged at 12 000 rpm for 20 min at 4 °C. The enzymatic activities as well as the protein contents are assayed in this extract. The measurement was carried out in a volume of 3 mL containing the phosphate buffer (50 mM; pH = 6.5), guaiacol (50 mM), H_2_O_2_ (2 %) and 100 μL of enzymatic extract. Enzyme activity was monitored by spectrophotometry at 470 nm.

The CAT activity is determined according to the method described by Beers and Sizer (1952). Hundred milligrams of fresh plant material were ground in a mortar with 1.5 mL of phosphate extraction buffer (50 mM; pH = 7). The ground material is then centrifuged at 12 000 rpm for 5 min at 4 °C. The enzymatic activities as well as the protein contents are assayed in this extract. For a final volume of 1 mL, a reaction mixture consisting of 50 µL of supernatant and 950 µL of H_2_O_2_ reagent (20 mM) was prepared. Absorbance is monitored at 240 nm.

### Drought Stress Indexes

Drought Susceptibility Index (DSI) and Stress Tolerance Index (TSI) were calculated based on DM (%) using the following equations (Fischer and Maurer, 1978; Golbashy *et al*., 2010; Grzesiak *et al*., 2012):


$$\begin{array}{l} DSI=\;\;\;\frac{1-\;\;\;(D/C)}{1-(xD/xC)} \end{array}$$

$$\begin{array}{l} TSI=\frac{CD}{xC} \end{array}$$

where *C*, *D* are dry matter of above ground part of the plant (DM) in control (*C*) and drought (*D*) treatments, respectively. *xC*, *xD* are average values for all examined genotypes of dry mater of aboveground part of the plant (DM) in control (*C*) and drought (*D*) treatments, respectively.

### Statistical analysis of the data

For statistical analysis, R v.3.6.1 was used (Core Development Team R, 2020). The effect of drought stress treatments on the studied genotypes was determined by a multivariate analysis of variance (MANOVA) test. A *post hoc* analysis was set up using Tukey’s multiple comparison test. All measured variables were used for the principal component analysis (PCA) which was carried out by using FactoMineR (Lê *et al*., 2008) and Factoextra (Kassambara and Mundt, 2017) libraries. To normalize data and to study the complex genotypic variability under water stress, the results were presented as Log_2_(Trait Fold Change (TFC)) = Log_2_*C* − Log_2_*D*, where *C* represents control value (100 % FC), and *D* refers to drought stress treatments (50 or 25 % FC).

In addition, an agglomerative hierarchical clustering, using unweighted pair group method with arithmetic mean (UPGMA) method, was performed to classify the genotypes according to their tolerance.

## Results

### Effect of drought stress on morpho-physiological traits and biochemical traits

In this study, 16 morpho-physiological and biochemical traits were used to screen drought stress tolerance of durum wheat genotypes. All those parameters were under the significant effect of genotype × water stress treatments (G × T) **[**see Supporting Information—Table S1**]**.

The APL was significantly reduced by water stress for most of the studied genotypes, except Hedhba, Jneh Khotifa and Hmira **[****see Supporting Information—Table S1****]**. The highest rate of decrease was observed under 25 % FC for the genotype Chili (30.9 %) compared to the control, while the Jneh Khotifa was the least impacted.

Drought stress led to the reduction of LA of 22.9 and 37.2 % under 50 and 25 % FC, respectively. The highest decrease was observed under severe stress (25 % FC) for Hmira (55.5 %), Chili (46.9 %) and Karim (40.1 %) **[****see Supporting Information—Table S1****]**.

Data showed that leaf temperature (LT) was not affected by water stress at early growth stage as for Aouija, Hedhba, Jneh Khotifa, Chili and Karim **[****see Supporting Information—Table S1****]**.

Drought stress led to an accumulation of phenolic compounds that was proportional to the severity of water stress. The highest increase of phenolic compounds was noted for Mahmoudi under 25 % FC with a 5-fold increase compared to control **[****see Supporting Information—Table S1****]**.

Soluble sugars changes were significantly (*P* < 0.001) affected by treatments **[****see Supporting Information—Table S1****]**. Maximum SSC increase under severe stressed conditions (25 % FC) was observed in Mahmoudi, Swabaa Aljia and Jneh khotiffa with a sugar content increase about 15-fold **[****see Supporting Information—Table S1****]**. These results were in accordance with Chaudhari *et al*. (2017) who observed that the soluble carbohydrate concentration in well-watered wheat plants was lower than that of stressed plants.

PC was significant (*P* < 0.001) under the interaction between genotypes and treatments **[****see Supporting Information—Table S1****]**. The proline accumulation increased two times under high stress treatment (25 % FC). The maximum increase in PC was noticed in Mahmoudi content under 25 % FC **[****see Supporting Information—Table S1****]**.

**Figure 1.**
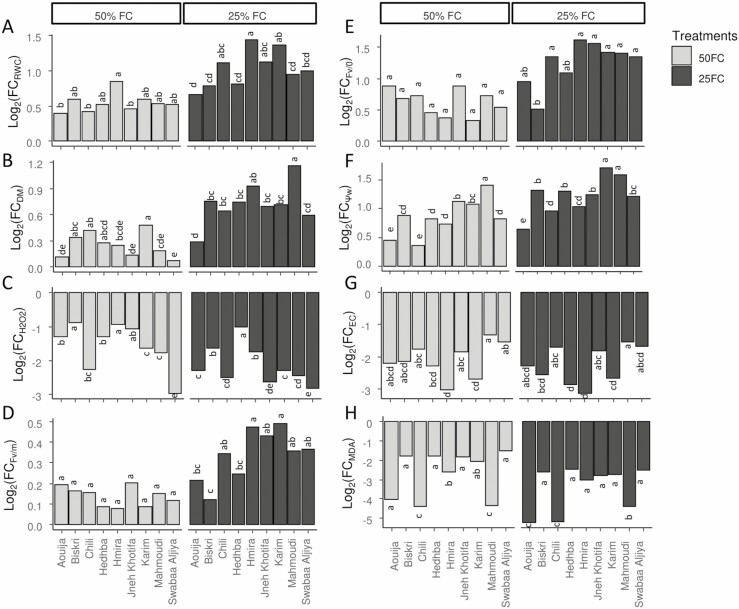
Log_2_(TFC) (*C* − *D*) = Log_2_*C* − Log_2_*D*, where *C* represents control value (100 % FC), and *D* refers to drought stress treatments (50 or 25 % FC) of eight traits of the nine studied genotypes. RWC: relative water content, DW: dry matter, H_2_O_2_: H_2_O_2_ content, F_v/m_: the maximum fluorescence efficiency, F_v/o_: leaf photosynthetic capacity, Ψ_w_: leaf water potential, EC: electrolyte conductivity, MDA: malondialdehyde content.

The variation of Log_2_(TFC) of eight traits of the nine studied genotypes under three irrigation treatments, are presented in Fig. 1. Positive Log_2_(TFC)_ (*C*−*D*)_ values indicate a decrease in the trait value under drought stress (D) compared to control plants (C). Negative values of Log_2_(TFC)_ (*C*−*D*)_ indicate an increase in the trait value. The results showed that all tested genotypes tend to decrease their RWC under 50 % FC mainly for Aouija followed by Jneh Khotifa and Chili (Fig. 1A). Under 25 % FC, the RWC decreased significantly compared to the control plants for all the genotypes. Moreover, the lowest decrease was observed in Aouija, Biskri and Hedhba. The decrease in RWC was not significant between 50 and 25 % FC for Biskri and Hedhba (Fig. 1A). Likewise, the DM rate was greatly reduced by the drought stress (Fig. 1B). Under 50 % FC, the genotype Karim was the most sensitive to drought FC with 28.4 % decrease. Under 25 % FC, the genotype Mahmoudi showed a decrease of 55 % compared to control. In addition, a remarkable increase was seen in the H_2_O_2_ content under drought stress (Fig. 1C). Under 50 % FC, the lowest rise was observed for Biskri (80 %) and Hmira (81.81 %). While under 25 % FC, Hedhba showed the lowest increase (91.67 %) in H_2_O_2_ content (Fig. 1C).

The F_v/o_ and the F_v/m_ decreased significantly (*P* < 0.001) under drought stress **[****see Supporting Information—Table S1****]**. The F_v/o_ and F_v/m_ ratios under drought stress decreased by 34.6 and 9 % under 50 % FC and by 56.45 and 20.58 % under 25 % FC, respectively (Fig. 1D and E). The lowest decrease of F_v/m_ was noted in Hedhba (5.8 %) under 50 % FC and in Biskri (8 %) under 25 % FC compared to the control treatment (Fig. 1D and E).

The Ψ_w_ was also reduced by the drought stress mainly under 25 % FC (135 %) than 50 % FC (85 %), compared to control. The genotype Aouija showed the lowest decrease (56 %) of Ψ_w_ under 25 % FC (Fig. 1F).

Drought stress drastically affected the membrane stability of the tested genotypes increasing by the way of the EC. The highest rise in EC was noted for Hmira and Karim under both drought stress treatments (Fig. 1G).

Besides, drought stress conditions stimulated MDA accumulation. Maximum increase was observed for Aouija and Chili under both levels of drought stress (Fig. 1H).

### Effect of drought stress on antioxidant enzyme activities

**Figure 2.**
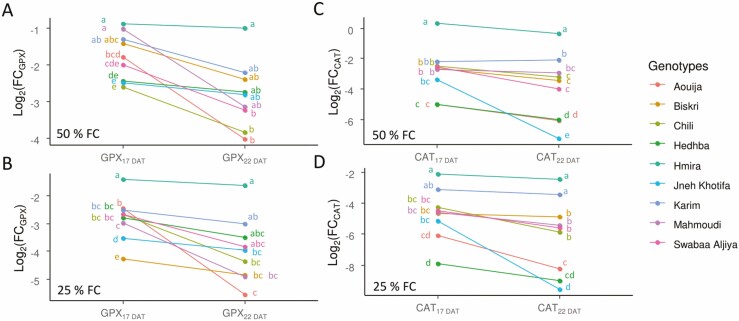
Variability of GPX and CAT activity content measured at 17th and 22nd days after drought of 50 and 25 % FC of nine durum wheat genotypes.

Data showed that GPX activity was higher when water stress levels increased from 50 to 25 % FC for most tested genotypes despite sampling dates (17 and 22 days after treatment (DAT)) (Fig. 2).

Under 50 % FC, the average GPX activity was higher at 22 DAT. While at 17 DAT, the highest increase was observed for Chili, Hedhba and Jneh Khotifa. Moreover, the lowest increase in GPX activity was observed for Hmira (85.25 %) on the 17 DAT. On 22 DAT, the GPX activity increased for all the genotypes. However, the most important increase was noted for Aouija, Chili and Swabaa Aljiya. The less increase was observed for Hmira (101.88 %) **[****see Supporting Information—Table S1****]**.

sUnder 25 % FC, all the studied genotypes increase their GPX activity on the 17 DAT relative to the control plants. The highest increase was observed in Biskri and Jneh Khotifa. Moreover, the lowest increase in GPX activity, on the 17 DAT, was noted for both. The lowest GPX activity was noted for Hmira with an increase rate equal to 167.32 % compared to the control treatment **[****see Supporting Information—Table S1****]**.

The results of this study showed that all the genotypes tend to increase their GPX activity on the 22 DAT compared to GPX activity on the 17 DAT. The rate of increase in GPX activity for most of the studied genotypes between the two sampling dates (17 DAT and 22 DAT) was higher under 25 % FC than under 50 % FC treatment (Fig. 2).

The results of CAT measured on nine genotypes under 50 % FC on the 17 DAT show that the CAT activity increased compared to the control treatment. The highest increase was measured in Aouija (Fig. 2C).

Under 50 % FC, the CAT activity increased on the 22 DAT compared to the 17 DAT in most of the tested genotypes. The highest increase was observed in Jneh Khotifa, Aouija and Hedhba (Fig. 2C). Moreover, the less increase was observed on Hmira with a rate of increase equal to 31.58 % compared to the control treatment **[****see Supporting Information—Table S1****]**.

On the other hand, under severe drought stress (25 % FC), the CAT activity increases on the 22 DAT compared to the CAT activity on 17 DAT (Fig. 2D). Indeed, on the 17 DAT, the increase in CAT activity was observed on Jneh Khotifa, Aouija and Hedhba. And the Log_2_(FC_CAT_) between 100 and 25 % FC values on the 17 DAT was equal, respectively, to −5.14, −6.07, and −7.9 (Fig. 2D).

### Relative contribution of measured parameters to plant biosynthesis under stress conditions

To evaluate the relative contribution of the measured parameters to wheat drought stress tolerance, estimated by DM, we proceeded to a multiple regression analysis using the step-by-step procedure (Stepwise). The dependent variable was chosen as DM at different water regimes (100, 50 and 25 % FC) for all the tested genotypes. The independent variables were those chosen to best fit the model (Table 1).

**Table 1.** Multiple linear regression analysis using stepwise procedure for dry matter (DM) content of nine durum wheat genotypes under three water regimes: 100, 50 and 25 % FC. Catalase activity (CAT) 17 and 22 days after stress treatment (CAT_17 DAT_ and CAT_22 DAT_), guaiacol peroxidases activity (GPX) 17 days after stress treatment (GPX_17 DAT_), soluble sugars content (SSC), chlorophyll Index (CI) and leaf area (LA).

Treatments (% FC)	Variables introduced	*R*²
DM 100	CAT_17 DAT_	0.5
	CAT_17 DAT_, SSC	0.73
	CAT_17 DAT_, SSC, MDA	0.81
	CAT_17 DAT_, SSC, MDA, RWC	0.84
DM 50	CI	0.4
	CI, SSC	0.68
	CI, SSC, CAT_17 DAT_	0.75
	CI, SSC, CAT_17 DAT_, H_2_O_2_	0.88
	CI, SSC, CAT_17 DAT_, H_2_O_2_, CAT_22 DAT_	0.92
	CI, SSC, CAT_17 DAT_, H_2_O_2_, CAT_22 DAT_, GPX_17 DAT_	0.94
DM 25	LA	0.65
	LA, SSC	0.75
	LA, SSC, CI	0.81

For DM at 100 % FC, the best model (*R*^2^ = 0.84) was obtained by the independent variables as CAT_17 DAT_, SSC, MDA and RWC. Under moderate water stress (50 % FC), DM was best determined (*R*^2^ = 0.94) by (CI), SSC, CAT_17 DAT_, H_2_O_2_, CAT_22 DAT_ and GPX_17 DAT_. Under severe water stress (25 % FC), the independent variables of LA, SSC and CI were among best model (*R*^2^ = 0.81).

The results of linear regression showed that SSC and CI are the most determining factors of DM under both moderate and severe water stress (50 and 25 % FC) (Table 1).

**Figure 3.**
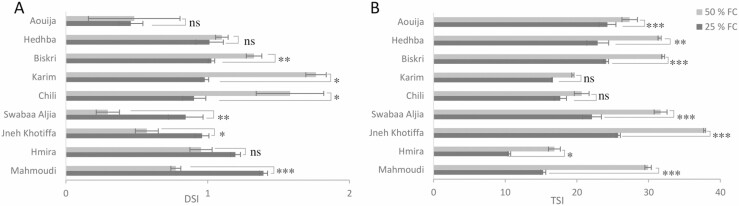
Drought tolerance indexes of nine genotypes under three treatments of irrigation, 100, 50 and 25 % FC based on their DM. The light grey colouration represents DSI or TSI determined for each genotype under 50 % FC stress. The dark grey colouring DSI or TSI calculated for each genotype under 25 % FC stress.

Under 50 % FC, the most important DSI based on the DM was observed for Karim and Chili, and the minimum was observed for Aouija (Fig. 3). Under 25 % FC, the most important DSI index was observed for Mahmoudi and Hmira, while lowest DSI was observed for Aouija. Results showed that Aouija is the most stable genotype based on DSI index with values below 1 under 50 % FC as well as under 25 % FC stress (Fig. 3). In addition, the TSI highest values were observed for Aouija, Hedhba, Biskri and Jneh Khotifa (Fig. 3).

### Genotypic clustering of durum wheat under water stress

**Figure 4.**
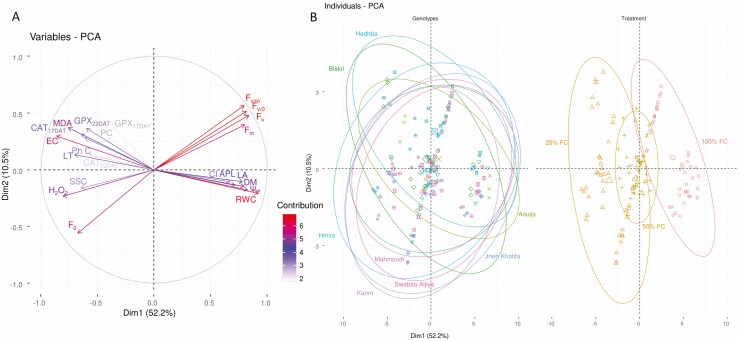
Principal component analysis of all the measured variables for the nine genotypes and three studied treatments (100, 50 and 25 % FC). (A) Visualization of the contribution of each variable to the variance of the model and the direction of the variation. (B) PCA visualization of individuals per genotype and per treatment. Ellipses with 95 % of confidence were represented to allow clustering among genotypes and treatments.

The PCA explained 64 % of the cumulative variance of measured parameters for durum wheat under water stress. The first principal component accounted for 52.3 % of the total variability; while the second component accounted for only 11.7 % (Fig. 4).

The traits that most contributed to PCA were chlorophyll fluorescence parameters, RWC and EC (Fig. 4A); while antioxidant enzymes (CAT and GPX), sugars and proline were the less PCA contribute traits (Fig. 4A).

In our study, the biplot derived from the PCA allowed the identification of four durum wheat genotype groups (Fig. 4B).

Indeed, the first and the second groups are formed by the different tested genotypes under 100 and 50 % FC. The third group is the genotypes Aouija, Hedhba and Biskri under 25 % FC. While the fourth group is constituted by the rest of tested genotypes under 25 % FC. PCA showed an ellipse of 95 % confidence of the genotypes Aouija, Hedhba and Biskri narrowing in the same direction (Fig. 4B).

**Figure 5.**
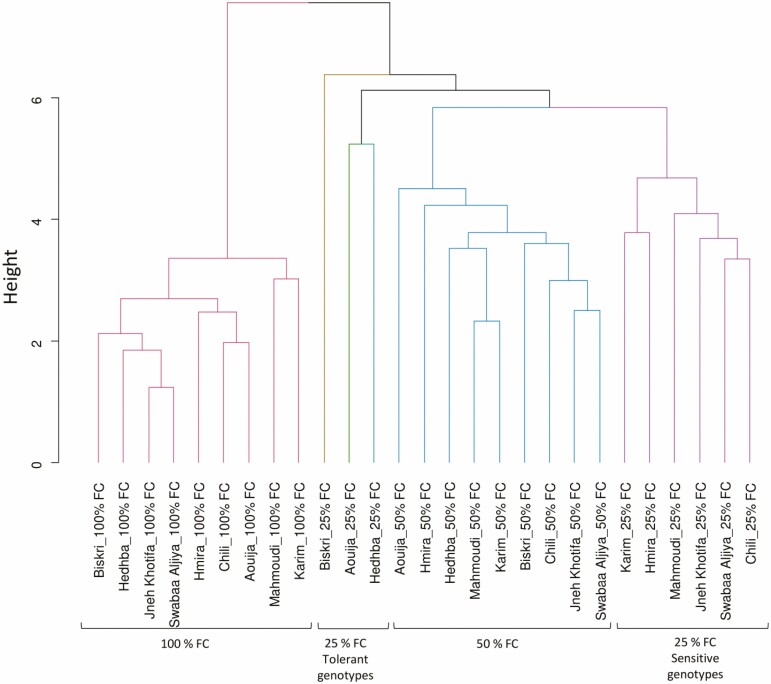
Dendrogram of the mean of all measured parameters and tested genotypes under three water stress treatments.

The UPGMA showed that the tested genotypes were clustered in three groups depending on the irrigation levels. However, genotypes under 25 % FC were clustered in two groups: one with the most drought-tolerant genotypes, clustered with the genotypes under 100 % FC, and the second cluster with the sensitive genotypes. The dendrogram clustering showed that drought-sensitive genotypes, i.e. Karim and Hmira were located together. Similar trend was observed for drought resistant genotypes, i.e. Aouija, Biskri and Hedhba (Fig. 5).

## Discussion

In the next years, climate change will continue to threaten the world’s food supply. In these circumstances, the development of new high-yielding varieties adapted to diverse environments is crucial (Mammadov *et al*., 2018). To improve drought tolerance, plant breeders must first select the potential germplasm that contains large genotypic variability for drought tolerance (Baenziger, 2016).

For this purpose, 16 characters were used to assess the variability of 9 durum wheat genotypes under water stress conditions applied at the early juvenile growth stage. In fact, the early growth stages affect the grain yield because the photosynthetic reserves accumulated until flowering provided approximately 57 % of the final grain yield (Gallagher *et al*., 1976).

The MANOVA revealed significant differences among tested genotypes as well as for the genotype × treatment interactions for all assessed traits. The same findings were reported by Khodadadi *et al*. (2011) and Mwadzingeni *et al*. (2016).

The use of PCA as a key solution for integrating all measured parameters to screen for drought stress has been successfully used in many species, including common bean (Ali *et al*., 2019), bread wheat (Ghasemi and Farshadfar, 2015) and the durum wheat (Kacem *et al*., 2017; Quagliata *et al*., 2023).

PCA allowed us to identify the traits that contributed most to the total variance. These traits were RWC, DM, H_2_O_2_ content, chlorophyll fluorescence parameters (*F*_v/m_ and *F*_v/o_), Ψ_w_, EC and MDA. A significant association between CI, chlorophyll fluorescence parameters (*F*_v/m_ and *F*_v/o_), RWC, APL, DM and water potential parameter was observed as previously in Grzesiak *et al*. (2019). Furthermore, a strong and positive relationship among antioxidant system, enzymatic activities (CAT and GPX), non-enzymatic content (phenolic compounds) and EC illustrates production of ROS in response to changes in membrane stability, which triggers production of antioxidants (Sheoran *et al*., 2015; Caverzan *et al*., 2016). In addition, PCA showed that landraces Aouija, Biskri, Hedhba and Jneh Khotifa were the most tolerant to drought stress genotypes, whereas the variety Karim was the most sensitive. Like related wild species (Maxted *et al*., 2008), landraces (Zeven, 1998; Ouaja *et al*., 2021) are considered a valuable source of germplasms for the breeding of drought-tolerant genotypes (Al Khateeb *et al*., 2017; Placido *et al*., 2020). These landraces exhibit a broader genetic background that results from both natural and farmer selection (Belay *et al*., 1995). Thus, using landraces such as Aouija, Biskri, Hedhba and Jneh Khotifa could be a way to improve wheat varieties exhibiting good nutritional characteristics and yield potential, notably under drought stress conditions (Al Khateeb *et al*., 2017).

### Effect of drought stress on plant water status and photosynthesis activity

RWC is one of the most important physiological traits to check the cell hydration and it’s probably the most meaningful measure of plant water status under water deficit (Silva *et al*., 2007; Qayyum *et al*., 2021).

Under water stress conditions, the RWC decreases independently of wheat’s growth stage. In this sense, Bentahar and Ykhlef (2017) observed a significant reduction in RWC only under severe stress (20 % FC) in potted durum wheat under semi-controlled conditions.

The decrease in RWC of sensitive genotype seedlings leaves could be attributed to a higher transpiration rate, due to the rapid loss of water before the stomata close, than water uptake compared to tolerant genotypes (Chaves *et al*., 2009; Flexas *et al*., 2009; Osakabe *et al*., 2014; Mondini and Pagnotta, 2015). Under rising water shortages, plant species lose water mainly by transpiration and then they tend to control their stomatal closure. In addition, many studies showed that stomatal closure is a genotypic depending component (Hajiboland *et al*., 2015; Ahmad *et al*., 2018; El *et al*., 2019).

Our results were similar to the findings of Pour-Aboughadareh *et al.* (2017) and Reza Beighi and Bijanzadeh (2020), indicating that RWC can be used as an important secondary trait for selecting wheat genotypes under drought conditions. Indeed, plants maintaining RWC under drought conditions are meant to be resistant (Ali *et al*., 2019; Karimpour, 2019; Sattar *et al*., 2019). Our data showed that Aouija, Jneh Khotifa and Chili showed the ability to maintain the highest RWC under drought stress conditions.

Chlorophyll Index decreased significantly compared to unstressed plants for most of the tested genotypes. Reduction in CI due to drought stress has been documented by many studies (Alachew *et al*., 2016; Chen *et al*., 2016; Khayatnezhad and Gholamin, 2021).

Besides, chlorophyll fluorescence analysis revealed that drought results in decreased electron transfer to the PSII reaction centre due to variations in energy absorption, trapping, electron transport and dissipation per section, resulting in a reduced photosynthetic efficiency of PSII (Stirbet *et al*., 2018; Khatri and Rathore, 2019). According to ŽIVČÁK *et al*. (2014), water stress progressively decreases PSII electron transport and increases non-photochemical quenching in wheat leaves that supports the role of alternative sink electrons (of PSII or PSI) and the flow of cyclic electrons for photo-protection of PSII and PSI, which also generate the ATP needed to cope with water stress conditions.

Furthermore, F_v/m_ and F_v/o_ are considered important parameters for assessing the integrity of the internal mechanism within a leaf during the photosynthetic activities and are considered as accurate methods for screening plant tolerance to drought stress (Sharma *et al*., 2015; Lee *et al*., 2016). Like other physiological traits, these parameters are highly affected by drought stress treatments **[****see Supporting Information—Table S1****]**. A reduction in F_v/m_ by water deficit suggests a possible inhibition of PSII photochemistry that may be due to inadequate energy relocation from the light-harvesting chlorophyll complex to the reaction centre (Ahmed *et al*., 2013). The minimum decrease of F_v/o_ and F_v/m_ was observed for Biskri and Aouija indicating that a higher defensive ability for PSII is a key resistance mechanism for wheat landraces, which agrees with previous studies for drought-treated durum wheat accessions (Hairat and Khurana, 2015). Those genotypes (Biskri and Aouija) are more tolerant to drought stress and could be an opportunity for plant breeders as potential sources of drought resistance.

### Osmotic adjustment

Dehydration of plant tissues impairs various physiological processes, especially the changes in leaf water potential (Grzesiak *et al*., 2019). The same results were recorded in our assay, where Ψ_w_ decrease was correlated with drought stress treatments.

Osmotic adjustment allows the plant to maintain turgor pressure at low water potential, which is important for maintaining metabolic functions (Izanloo *et al*., 2008; Bhutto *et al*., 2023).

We observed an increase in leaf’s PC under drought conditions as previously reported (Nowsherwan *et al*., 2018; Ali *et al*., 2019). Proline accumulation is involved in maintaining plant growth under drought conditions through the osmo-protective and developmental functions of amino acids (Batista-Silva *et al*., 2019). The results showed that the genotypes Mahmoudi and Karim have the most important proline accumulation. High capacity of proline accumulation under water deficit is commonly associated with high drought stress tolerance (Bilal *et al*., 2015; Quagliata *et al*., 2023). However, the relationship between proline accumulation and enhanced or reduced tolerance to stress is still controversial in the literature, having been reported correlation of higher proline concentrations with both higher and lower stress tolerance (Arteaga *et al*., 2020). This amino acid is also involved in scavenging ROS, thus maintaining protein stability and pH homeostasis in the cytoplasm (Bandurska *et al*., 2017).

Under drought stress conditions, plants accumulate other osmotically active substances as sugars (Sami *et al*., 2016; Basu *et al*., 2016; Hussein *et al*., 2022; Chunyan *et al*., 2022). Our data showed a substantial increase in this solute correlated with water stress levels as previously reported (Sedaghat *et al*., 2020; Adrees *et al*., 2020). The increase in SSC was higher under 25 % FC than 50 % FC treatments and this trend was more pronounced in Jneh Khotifa than in the other tested genotypes. In fact, sensitive plants showed less of an increase in SSC than did tolerant plants (Abid *et al*., 2018*a*).

### Cell membrane stability

Our data showed that electrolyte leakage progressively amplified with increasing drought severity. These results are in agreement with a set of previous studies (Chaudhari *et al*., 2017). Similar to our results, Hairat and Khurana (2015) and Pour-Aboughadareh *et al*. (2017) reported that different species have different effect on drought stress as some are less affected than others, while depending on their thylakoid membrane stability under drought stress. A strong correlation between cell membrane stability with growth and field performance of wheat seedlings has been previously reported (Bajji *et al*., 2001), which resembles our results.

### Oxidative homeostasis

Photosynthetic activity is reduced under drought stress leading to ROS accumulation as a result of photosynthetic electron transport reactions under the situations of saturated electron flow (Reddy *et al*., 2004). Drought is among an excessive accumulation of H_2_O_2_ and of MDA, a marker to measure the amplitude of oxidative damage in the stressed conditions (Porcel and Ruiz-Lozano, 2004).

Higher H_2_O_2_ production under drought stress treatments might be associated with photosynthetic damage. The highly reactive H_2_O_2_ damages the photosynthetic system, oxidizes proteins, lipids, nucleic acids and carbohydrates, and damages cell membranes (Reddy *et al*., 2004; Sabra *et al*., 2012). Our results showed that higher MDA concentrations in the drought-stressed plants were associated with higher H_2_O_2_ content, especially under severe drought-stress conditions. Indeed, the maximum values of H_2_O_2_ and MDA were found under limited water levels (50 and 25 % FC) in the genotype Chili (Fig. 1C).

Changes in antioxidant enzyme activities in response to water deficit are meant to maintain the fine balance between the generation and detoxification of ROSs at the intracellular level (Ahmadi *et al*., 2018*b*; Zhao *et al*., 2020). Tolerant genotypes are characterized by high antioxidant capacity to alleviate oxidative damage and maintain structural integrity of cell components (Ahmadi *et al*., 2018*a*). Plants have a developed antioxidative system that uses the enzymes superoxide dismutase, ascorbate peroxidase, catalase, GPX, peroxiredoxins, monodehydroascorbate reductase, dehydroascorbate reductase and glutathione reductase to reduce and eliminate ROS-mediated oxidative damage (Soares *et al*., 2019). However, changes in antioxidant enzyme activities under drought stress are dependent on plant species, cultivar, stress intensity and duration (Chmielewska *et al*., 2016; Soni *et al*., 2017). Indeed, the rate of increase in GPX activity for most of the studied genotypes between the two sampling dates (17 DAT and 22 DAT) was higher under 25 % FC than under 50 % FC treatment.

During the early days of drought, plants showed a rapid increase in CAT and GPX activities as it scavenged H_2_O_2_, produced after the dismutation of O_2_^−^ by superoxide dismutase (Movludi *et al*., 2014). It is well-evident that the generation of H_2_O_2_ is the primary signal for the initiation of antioxidant enzyme activities (Abid *et al*., 2018*a*) and therefore is an essential component of the ROS-mitigation system.

Higher CAT and GPX activities with lower H_2_O_2_ accumulation in Biskri and Hedhba plants indicate the existence of an improved redox defense potential to drought stress (Fig. 1).

During the early days of stress, the activities of both CAT and GPX increased 17 DAT, above all, under 25 % FC, for most of the tested genotypes except Karim and Hmira. Those two genotypes have the lowest CAT and GPX activities (Fig. 2). These contexts may give the explanation for the lower capability of Karim and Hmira plants to build and maintain a high activity of antioxidants under drought stress as compared to Biskri and Hedhba plants. In contrast, the plants under Biskri and Hedhba displayed enhanced enzymatic activities of CAT and GPX and consequently lower H_2_O_2_ production as compared to Hmira and Karim plants. Moreover, GPX activity was more important than CAT activity when the drought stress treatments reached 22 DAT for most of the tested genotypes. CAT and GPX of landraces wheat genotypes showed greater activities in response to water stress than cultivated wheats at seedling stage (Ahmadi *et al*., 2018*b*).

### Durum wheat drought stress selection criteria

A several studies have recently identified biomass and antioxidant system as proxies for selection of wheat genotypes at the young seedling stage under drought (Mickky and Aldesuquy, 2017; Ahmadi *et al*., 2018*b*). During water deficit, plants reduce their vegetative growth by reducing cell proliferation and expansion to maintain reserves and thus sustain survival as growth adjustment mechanism (Nahar *et al*., 2015).

Among the various stress tolerance indices, TSI has been suggested as a suitable selection criterion during early growth because this parameter enables us to identify individuals with high performance and stress-tolerant potential under adverse conditions (Pour-Aboughadareh *et al*., 2017). Abid *et al*. (2018*b*) and Aberkane *et al*. (2021) used the Drought-Tolerant Index to investigate drought tolerance in durum wheat genotypes. The genotypes Aouija, Hedhba, Biskri and Jneh Khotifa had the highest TSI. Similarly, high TSI is associated with a limited decline in RWC, DM and chlorophyll content in response to water deficit (Pour-Aboughadareh *et al*., 2017).

Drought Susceptibility Index is another selection index for contrasting genotypes under drought stress conditions. In fact, the genotypes, which carry the lowest DSI values are marked as drought-tolerant wheat (Mwadzingeni *et al*., 2016). In our case, the lowest DSI value was observed under severe stress in Aouija.

Cluster analysis with all measured parameters and calculated indexes under three levels of irrigation (100, 50 and 25 % FC) showed that the genotypes Aouija, Biskri and Hedhba could be used in breeding programmes as potential tolerant genotypes.

## Conclusions

This study showed that drought stress had negative effects on most durum wheat genotypes. Data revealed that RWC, CI, chlorophyll fluorescence parameters, water potential, EC, H_2_O_2_, MDA and DM are involved in drought tolerance and can be used as selective traits for screening durum wheat genotypes at seedling stage. Based on DSI and TSI under drought stress conditions, the landraces Aouija, Biskri and Hedhba showed drought stress tolerance aptitude. However, Karim and Hmira were drought sensitive genotypes.

Our results identified several tolerant genotypes that responded well to water stress and could be candidates for further studies of molecular mechanisms underlying other physiological and biochemical changes and subsequent yield improvements under drought stress. In addition, the identified landrace durum wheats represent valuable genetic resources that can be used for isolation of new drought resistance-associated genes or alleles to develop new wheat varieties that produce high yields in drought-prone areas.

In terms of perspective, it is interesting to complete this study with the tools of molecular genetics. Indeed, the search for molecular markers, the study of the genetic determinism and the heritability of identified characters could lead to the screening of durum wheat genotypes tolerant to drought stress, as good indicators of water stress tolerance are needed to facilitate the use of these traits in breeding programs. Moreover, these results need to be confirmed at later stages of development.

## Supporting Information

The following additional information is available in the online version of this article —

**Table S1.** Means of all measured traits as: the aerial part length (APL), leaf area (LA), proline content (PC), the soluble sugars content (SSC), initial fluorescence (F_0_), maximum fluorescence (F_m_), variable fluorescence (Fv), quantum yield (F_v/m_) and reaction center activity (F_v/o_), the leaf temperature (LT), the chlorophyll index (CI), the malondialdehyde content (MDA), phenolic compounds content (Ph.C), Guaiacol peroxidases activity 17 and 22 days after stress treatment (GPX_17 DAT_ and GPX_22 DAT_), catalase activity 17 and 22 days after stress treatment (CAT_17DAT_ and CAT_22DAT_), the relative water content (RWC), dry matter rate (DM), the hydrogen peroxide content (H_2_O_2_), the plant water potential (ψ_w_) and the electrolytic conductivity (EC), for the nine durum wheat genotypes conducted under three water regimes: 100%, 50% and 25% FC. ANOVA (up) and MANOVA (bottom) results are represented. Tukey’s test was performed to compare treatment’s mean.

plad022_suppl_Supplementary_Table_S1

plad022_suppl_Supplementary_Data

## Notes

**Plants, Ecosystems & Climate. Guest editor:** Mary Heskel
