# Correction to: The opportunity of using durum wheat landraces to tolerate drought stress: screening morpho-physiological components

**DOI:** 10.1093/aobpla/plae042

**Published:** 2024-08-14

This is a correction to: Latifa Chaouachi, Miriam Marín-Sanz, Zayneb Kthiri, Sameh Boukef, Kalthoum Harbaoui, Francisco Barro, Chahine Karmous, The opportunity of using durum wheat landraces to tolerate drought stress: screening morpho-physiological components, *AoB PLANTS*, Volume 15, Issue 3, June 2023, plad022, https://doi.org/10.1093/aobpla/plad022

A typographical error affecting the first equation in the section ‘Morpho-physiological traits’ has been corrected. ‘FW’, originally published as the numerator, has been moved to the denominator position; ‘DW’, originally the denominator, has been moved to the numerator position. Stated results are not affected.

