# Supplementary material for: The opportunity of using durum wheat landraces to tolerate drought stress: screening morpho-physiological components
Source: AoB Plants. 2023 May 5;15(3):plad022. doi: 10.1093/aobpla/plad022 (PMC10205476; doi:10.1093/aobpla/plad022)
Supplement: plad022_suppl_Supplementary_Table_S1 [file plad022_suppl_supplementary_table_s1.pdf]

Supplementary Table 1. Means of all measured traits as: the aerial part length (APL), leaf area (LA), proline content (PC), the soluble sugars content (SSC), initial fluorescence (F_0), maximum fluorescence (F_m), variable fluorescence (F_v), quantum yield ($F_{v/m}$) and reaction center activity ($F_{v/o}$), the leaf temperature (LT), the chlorophyll index (CI), the malonedialdehyde content (MDA), phenolic compounds content (Ph.C), Guaiacol peroxidases activity 17 and 22 days after stress treatment (GPX_{17DAT} and GPX_{22DAT}), catalase activity 17 and 22 days after stress treatment (CAT_{17DAT} and CAT_{22DAT}), the relative water content (RWC), dry matter rate (DM), the hydrogen peroxide content (H_2O_2), the plant water potential (ψ_w) and the electrolytic conductivity (EC), for the nine durum wheat genotypes conducted under three water regimes: 100%, 50% and 25% FC. ANOVA (up) and MANOVA (bottom) results are represented. Tukey's test was performed to compare treatment's mean. *, $P < 0.05$; **, $P < 0.01$; ***, $P < 0.001$; NS: Not Significant.

Genotypes (G)	Treatments (T)	APL		LA		PC		SSC		F_v		F_0		Fm		LT		CI		MDA		Ph.C	
Mahmoudi	100 % FC	36.83	a	11.13	a	0.13	a	0.01	a	1078.67	a	231.67	a	1310.33	a	21.4	a	32.81	a	0.02	a	0.12	a
	50 % FC	30.33	b	8.97	b	0.36	b	0.11	b	796	b	284.33	b	1080.33	b	23.03	a	29.67	b	0.35	b	0.36	b
	25 % FC	27.17	c	8.18	b	1.09	c	0.15	c	620.33	c	347.33	c	967.67	c	25.67	b	19.97	c	0.36	b	0.59	c
Hmira	100 % FC	44.17	a	14.14	a	0.65	a	0.04	a	997.33	a	235.67	a	1233	a	20.83	a	36.37	a	0.03	a	0.11	a
	50 % FC	35.83	ab	8.99	b	0.78	a	0.11	b	808.67	b	254.67	a	1063.33	b	26.67	b	20.57	b	0.19	b	0.19	b
	25 % FC	32.83	b	6.29	b	0.78	a	0.12	b	516.33	c	378.33	b	894.67	c	26.67	b	16.1	c	0.25	c	0.19	b
Jneh Khotiffa	100 % FC	40.67	a	16.19	a	0.31	a	0.01	a	1030	a	243.67	a	1273.67	a	23.43	a	41.33	a	0.07	a	0.12	a
	50 % FC	39.67	a	12.83	b	0.66	b	0.15	b	747	b	298.67	b	1045.67	b	24.1	a	38.33	b	0.25	b	0.24	b
	25 % FC	31.83	b	9.89	b	0.74	b	0.24	c	546.33	c	324.67	b	871	c	25.3	a	35.87	b	0.49	c	0.45	c
Swabaa Aljia	100 % FC	43	a	14.78	a	0.48	a	0.02	a	1005.67	a	252	a	1257.67	a	23.23	a	41.43	a	0.05	a	0.15	a
	50 % FC	38.5	b	11.7	b	0.92	ab	0.24	b	904	b	287.67	ab	1191.67	b	24.57	ab	39.37	a	0.14	b	0.14	a
	25 % FC	35.5	c	9.28	c	0.78	b	0.29	c	585	c	325.67	b	910.67	c	25.43	b	29.8	b	0.29	c	0.41	b
Chili	100 % FC	51.83	a	17.1	a	0.65	a	0.04	a	1075	a	240.67	a	1315.67	a	21.53	a	36.4	a	0.02	a	0.25	a
	50 % FC	38.33	b	13.69	b	0.66	a	0.07	b	807.33	b	276.33	b	1083.67	b	23.57	a	30.83	ab	0.38	b	0.18	b
	25 % FC	35.83	c	9.08	c	0.64	a	0.16	c	622	c	353.33	c	975.33	c	24.47	a	26.7	b	0.65	c	0.42	c
Karim	100 % FC	35.67	a	12.42	a	0.26	a	0.01	a	909	a	290.33	a	1199.33	a	22.9	a	36.77	a	0.04	a	0.32	a
	50 % FC	30	b	9.09	b	0.64	b	0.08	b	704.33	b	270.67	a	975	b	24.33	a	30.23	b	0.15	b	0.43	b
	25 % FC	24.83	c	7.44	b	1.11	c	0.09	b	458	c	337.33	b	795.33	c	25.57	a	23.67	c	0.24	c	0.34	a
Biskri	100 % FC	37.17	a	14.07	a	0.66	a	0.03	a	995.67	a	241.67	a	1237.33	a	22.37	a	37	a	0.08	a	0.21	a
	50 % FC	31	b	9.48	b	0.7	a	0.29	b	709.67	b	284.67	a	994.33	b	26.23	b	24.07	b	0.27	b	0.27	ab
	25 % FC	27.5	c	8.84	b	0.7	a	0.29	b	708.33	b	260.67	a	969	c	26.23	b	20.03	c	0.47	c	0.28	b
Hedhba	100 % FC	44.83	a	15.74	a	0.58	a	0.02	a	1136.67	a	224	a	1360.67	a	23.53	a	39.97	a	0.12	a	0.23	a
	50 % FC	32	b	12.14	b	1.07	a	0.13	b	960.67	a	283	a	1243.67	ab	24.27	a	39.27	a	0.41	b	0.42	b
	25 % FC	34.5	b	10.32	b	1.07	a	0.13	b	807.33	b	337	b	1144.33	b	24.27	a	37.43	b	0.66	c	0.68	c
Aouija	100 % FC	49.33	a	19.39	a	0.46	a	0.03	a	1016.67	a	204	a	1220.67	a	21.83	a	45.3	a	0.02	a	0.14	a
	50 % FC	41.83	b	14.09	b	0.69	b	0.16	b	737.33	b	298	b	1035.33	b	23.2	a	38.63	b	0.24	b	0.33	b
	25 % FC	38.17	c	13.92	b	0.93	c	0.24	c	634.67	c	269.33	b	904	c	23.27	a	34.77	c	0.55	c	0.5	c
Genotypes (G)		***		***		***		***		***		NS		***		***		***		***		***	
Treatments (T)		***		***		***		***		***		***		***		***		***		***		***	
G * T		***		**		***		***		***		*		***		**		***		***		***	

Genotypes (G)	Treatments (T)	GPX 17 DAT		GPX 22 DAT		CAT 17 DAT		CAT 22 DAT		RWC		DM		H_2O_2		$F_{v/m}$		$F_{v/0}$		ψ_w		EC	
Mahmoudi	100 % FC	0.043	a	0.026	a	0.006	a	0.006	a	94.72	a	33.1	a	0.04	a	0.83	a	4.84	a	-5.33	a	22.38	a
	50 % FC	0.088	b	0.213	b	0.040	b	0.040	b	65.76	b	29.02	b	0.15	b	0.74	b	2.93	b	-14.21	b	55.38	b
	25 % FC	0.342	c	0.735	c	0.154	c	0.154	c	49.18	c	14.83	c	0.24	c	0.65	c	1.82	c	-16.13	c	65.61	b
Hmira	100 % FC	0.136	a	0.160	a	0.015	a	0.015	a	88	a	25.33	a	0.11	a	0.81	a	4.33	a	-14.84	a	10.65	a
	50 % FC	0.251	b	0.323	b	0.011	a	0.011	a	49.06	b	21.34	a	0.2	b	0.77	a	3.33	b	-24.58	b	86.29	b
	25 % FC	0.363	c	0.496	c	0.061	b	0.061	b	32.47	c	13.3	b	0.36	c	0.58	b	1.4	c	-30.46	c	93.9	c
Jneh Khotiffa	100 % FC	0.030	a	0.036	a	0.005	a	0.005	a	91.78	a	36.53	a	0.05	a	0.82	a	4.64	a	-9.54	a	16.3	a
	50 % FC	0.169	b	0.254	b	0.032	b	0.032	b	66.53	b	33.21	b	0.11	b	0.71	b	2.52	b	-20.8	b	58.39	b
	25 % FC	0.351	c	0.560	c	0.109	c	0.109	c	42.03	c	22.63	c	0.34	c	0.61	c	1.59	c	-22.62	c	58.56	b
Swabaa Aljia	100 % FC	0.040	a	0.029	a	0.009	a	0.009	a	97.81	a	32.66	a	0.03	a	0.81	a	4.35	a	-10.13	a	17.6	a
	50 % FC	0.162	b	0.276	b	0.055	b	0.055	b	68.42	b	31.12	b	0.26	b	0.75	b	2.95	b	-17.98	b	51.34	b
	25 % FC	0.258	c	0.427	c	0.212	c	0.212	c	49.09	c	21.73	c	0.23	c	0.63	c	1.71	c	-23.47	c	57.26	b
Chili	100 % FC	0.045	a	0.034	a	0.007	a	0.007	a	91.67	a	29.75	a	0.05	a	0.82	a	4.71	a	-12.96	a	16.25	a
	50 % FC	0.273	b	0.287	b	0.036	b	0.036	b	68.56	b	22.28	b	0.22	b	0.74	b	2.85	b	-16.62	b	55.23	b
	25 % FC	0.289	b	0.418	c	0.125	c	0.125	c	42.4	c	19.08	b	0.26	c	0.65	c	1.84	c	-25.21	c	53.32	b
Karim	100 % FC	0.066	a	0.068	a	0.013	a	0.013	a	87.34	a	29.52	a	0.07	a	0.76	a	3.27	a	-8.12	a	10.24	a
	50 % FC	0.164	b	0.311	b	0.058	b	0.058	b	57.82	b	21.14	b	0.2	b	0.72	a	2.6	b	-17.13	b	66.17	b
	25 % FC	0.383	c	0.540	c	0.110	c	0.110	c	33.95	c	17.97	b	0.32	c	0.55	b	1.29	c	-26.71	c	64.49	b
Biskri	100 % FC	0.121	a	0.106	a	0.005	a	0.005	a	85.64	a	36.08	a	0.1	a	0.8	a	4	a	-8.23	a	14.84	a
	50 % FC	0.322	a	0.562	b	0.031	b	0.031	b	56.67	b	28.47	b	0.18	b	0.71	b	2.47	b	-15.23	b	66.25	b
	25 % FC	2.328	b	3.082	c	0.123	c	0.123	c	49.61	b	21.42	c	0.31	c	0.73	b	2.85	b	-20.6	c	86.55	b
Hedhba	100 % FC	0.118	a	0.114	a	0.002	a	0.002	a	81.55	a	35.02	a	0.12	a	0.84	a	5.15	a	-10.31	a	12.13	a
	50 % FC	0.641	b	0.760	b	0.046	b	0.046	b	56.77	b	28.9	b	0.28	b	0.79	b	3.76	b	-18.28	b	55.33	b
	25 % FC	0.826	c	1.300	c	0.346	c	0.346	c	46.86	b	20.98	c	0.23	c	0.71	c	2.42	c	-25.55	c	82.12	c
Aouija	100 % FC	0.084	a	0.075	a	0.003	a	0.003	a	96.88	a	30.84	a	0.04	a	0.83	a	4.83	a	-10.78	a	12.89	a
	50 % FC	0.289	b	1.222	b	0.105	b	0.105	b	73.76	b	28.48	b	0.11	b	0.72	b	2.63	b	-14.75	b	58.47	b
	25 % FC	0.459	c	3.524	c	0.222	c	0.222	c	61.07	c	25.24	c	0.21	c	0.71	b	2.51	b	-16.83	c	61.89	b
Genotypes (G)		***		***		***		***		***		***		***		***		**		***		***	
Treatments (T)		***		***		***		***		***		***		***		***		***		***		***	
G * T		***		***		***		***		***		***		***		**		*		***		***	
